# Effect of the Renin-Angiotensin-Aldosterone System Reactivity on Endothelial Function and Modulative Role of Valsartan in Male Subjects with Essential Hypertension

**DOI:** 10.3390/jcm10245816

**Published:** 2021-12-13

**Authors:** Jakub Jasiczek, Małgorzata Trocha, Arkadiusz Derkacz, Ewa Szahidewicz-Krupska, Adrian Doroszko

**Affiliations:** 1 Department of Cardiology, Provincial Specialized Hospital in Wroclaw, Kamienskiego 73a, 51-124 Wroclaw, Poland; j.jasiczek@gmail.com; 2 Department of Pharmacology, Faculty of Medicine, Wroclaw Medical University, Mikulicz-Radecki 2, 50-349 Wroclaw, Poland; malgorzata.trocha@umw.edu.pl; 3 Department of Internal Medicine, Occupational Diseases, Hypertension and Clinical Oncology, Faculty of Medicine, Wroclaw Medical University, Borowska 213, 50-556 Wroclaw, Poland; arkadiusz.derkacz@umw.edu.pl (A.D.); ewa.szahidewicz-krupsa@umw.edu.pl (E.S.-K.)

**Keywords:** renin angiotensin aldosterone system (RAAS), endothelial dysfunction, angiotensin receptor blockers (ARB), valsartan, asymmetric dimethylarginine (ADMA), nitric oxide (NO)

## Abstract

Background: The aim of the study was to evaluate the relationship between renin-angiotensin-aldosterone (RAA) system activity and reactivity, and the endothelial function profile in normotensive subjects (N), and in essential hypertensives (H), followed by analysis of the modulatory role of an angiotensin receptor blocker (ARB): valsartan, administered in the management of hypertension. Methods: A total of 101 male subjects were enrolled to the study: 31H and 70N. The nitric-oxide (NO) bioavailability (l-Arginine, asymmetric dimethylarginine (ADMA)), symmetric dimethylarginine (SDMA), endothelial vasodilative function (flow mediated dilation (FMD)), oxidative-stress markers (malonyldialdehyde (MDA), thiol index (GSH/GSSG), nitrotyrozine (*N*-Tyr)), and pro-inflammatory/angiogenic parameters (sICAM-1, sVCAM-1, PAI-1, sE-selectin, PAI-1, thromboxane -B2) were assessed at baseline, then after intravenous -l-arginine administration, which was repeated after the 4-day acetylsalicylic acid (ASA) administration (75 mg/24 h). In hypertensives, this whole protocol was repeated following 2 weeks of valsartan therapy. Results: No effect of valsartan and ASA on the flow-mediated vasodilation (FMD) and the NO bioavailability in hypertensives was observed. Administration of valsartan increased plasma renin activity (PRA), but without a decrease in the aldosterone levels. ASA treatment minimized the pre-existing differences between the groups, and increased the PRA in the N-subgroup with the highest ARR values. The blood concentrations of proinflammatory sICAM-1, sE-selectin, sVCAM-1, and PAI-1 were higher, whereas the anti-inflammatory 6-keto-PGF1 alpha level was lower in hypertensive subjects. The levels of angiogenic VEGF did not differ between groups. Conclusions: Our study does not confirm the modulative effect of valsartan on endothelial function. Normotensive men showed an increase in FMD after l-arginine administration, possibly indicating baseline impairment of the NO synthesis.

## 1. Introduction

The renin-angiotensin-aldosterone (RAA) system is among the most important contributors to the pathogenesis of cardiovascular disease, including hypertension. The action of Angiotensin II (Ang II) is mainly mediated by the type 1 Ang II receptor (AT1R) [1], which is expressed in numerous tissues, including the heart, kidney, brain, adrenal glands, blood vessels, and platelet surfaces [2]. The angiotensin receptor blockers (ARBs) are commonly used in the management cardiovascular disorders. Some studies indicate their pleiotropic effects, including those related to the function of the vascular endothelium [3].

**Figure 1:**
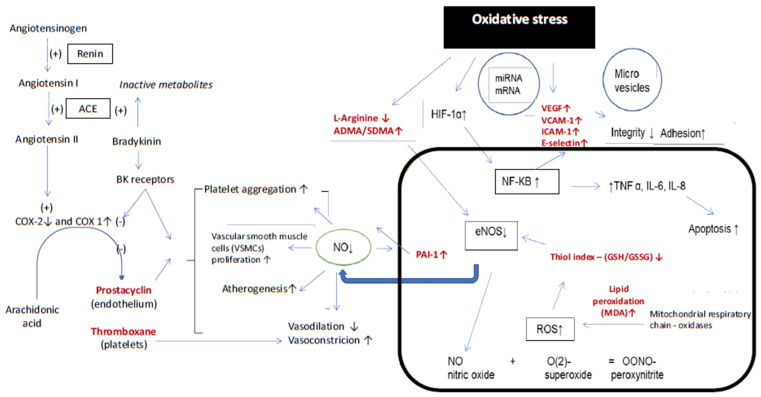
The links between the renin-angiotensin-aldosterone system, arachidonic acid cascade, endothelial dysfunction, and oxidative stress. The parameters analyzed in this study are marked in red. ADMA—asymmetric dimethylarginine, COX-cyclooxygenase, ACE—angiotensin converting enzyme, BK receptors—receptors for bradykinin, NO—nitric oxide, PAI-1—plasminogen activator inhibitor—1, MDA—malonyldialdehyde, GSH—reduced glutathione, GSSG- oxidized glutathione, eNOS—endothelial nitric oxide synthase, NF-KB—nuclear factor-kappa B, TNF α—tumor necrosis factor alpha, IL-6 and IL-8—interleukin 6 and 8, HIF 1α—hypoxia-induced factor alpha, ICAM-1—intracellular adhesion molecule, SDMA—symmetric dimethylarginine, VCAM-1—vascular cell adhesion molecule, VEGF—vascular endothelial growth factor.

The vascular endothelium, by producing numerous local and systemic substances, regulates the vascular tone, and maintains the local balance between pro- and anti-inflammatory, as well as pro- and anticoagulant factors [4]. It also interacts with circulating blood cells, and participates in the formation of new vessels [5]. Endothelial dysfunction occurs in the presence of an increased amount of reactive oxygen species (ROS), pro-inflammatory cytokines (ICAM-1, VCAM-1, E-selectin), and bacterial endotoxins, and in response to the presence of some cardiovascular risk factors. Due to reduced nitric oxide (NO) bioavailability, the endothelium loses the ability to control vascular tone, maintain the homeostasis, and limit the inflammatory reactions [6]. Prostanoids and, in particular, thromboxane (Tx)—a product of the arachidonic acid oxygenation in a reaction catalyzed by cyclooxygenase (COX), may participate in the pathogenesis of endothelial dysfunction induced by aldosterone. Its development is also possible during a stable blood pressure, and is limited by both aldosterone receptor blockade and inhibition of COX. Increased production of thromboxane (TxA2) is observed in aldosterone-induced endothelial dysfunction, suggesting an association between the cyclooxygenase (COX) pathway and the renin-angiotensin-aldosterone (RAA) system. Blocking the thromboxane (TP) receptor resulted in the inhibition of this dysfunction [7]. Interestingly, prostacyclin (PGI2), considered the major vasodilatory metabolite of arachidonic acid, has been shown to promote vasoconstriction by activating the TP receptor [8,9]. Prostanoids may promote endothelial dysfunction, partially reversible by acetylsalicylic acid (ASA) [10]. Therefore, it has been suggested that endothelial dysfunction can be minimized not only by limiting the RAA activation, but also by inhibiting the COX activity using selective or nonselective inhibitors [7]. Repeated attempts have been made to answer the question, and whether the effects of ARBs’ action (related to blocking the AT1 receptor) go beyond this mechanism. Up to date, the results of these studies are incomplete and contradictory [11,12,13,14]. Figure 1 presents conceptual links between the renin-angiotensin-aldosterone system, the arachidonic acid cascade, and oxidative stress leading together to the development of endothelial dysfunction, and promoting atherogenesis.

Hence, the aim of the study is to evaluate the effect of valsartan, an ARB, on endothelial function in male patients with isolated essential arterial hypertension, and the potentially modulative effect of the COX-1 blockade on its action. The relationship between the activity and reactivity of the RAA system and endothelial function was also analyzed—both in healthy male subjects, and those with essential arterial hypertension.

## 2. Materials and Methods

### 2.1. The Bioethics Statement, Protocol Approvals, and Patient Consents

The experiment was approved by the Local Bioethics Committee, and adhered to the principles of the Declaration of Helsinki and Title 45, U.S. Code of Federal Regulations, Part 46, Protection of Human Subjects (revised 13 November 2001, effective 13 December 2001). All participants provided their written consent to participate in the study. The written consent form was approved by the ethics committee.

### 2.2. Study Design and Groups Description

The study involved a total of 101 male subjects, at age of 18–40 years, including 31 patients with essential hypertension (H), and 70 healthy male volunteers—normotensives (N).

The hypertension was diagnosed strictly following the guidelines by the European Society of Hypertension and European Society of Cardiology (ESH and ESC). Each subject following several in-office and ambulatory measurements of blood pressure who had elevated values of blood pressure underwent the ABPM (ambulatory blood pressure monitoring for 24 h) to confirm the hypertension in cardiac ambulatory clinics. As the diagnosis was confirmed, the subjects wishing to participate in this study were hospitalized at the Department of Hypertension in order to exclude/confirm secondary hypertension (i.e., hormonal testing, imaging diagnosis, Epworth Sleepiness Scale (ESS) [15], and Berlin Questionaire [16], as well as sleep polygraphy for subjects at increased risk for sleep apnea). Only subjects with essential hypertension were included in the study group. The H population was recruited from cardiology ambulatory centers—including subjects with newly diagnosed hypertension. The normotensive group was formed by demographically matched normotensive, clinically healthy subjects wishing to undergo cardiovascular tests. The knowledge regarding this study was widespread among the local general practitioners as preventive cardiovascular examinations and screening tests for apparently healthy people. Subjects with white-coat hypertension, prehypertension, as well as with the secondary hypertension were excluded from the study. Exclusion criteria for both groups were: inability to provide an informed consent; lack of a detailed past medical history comorbidities list; confirmed secondary arterial hypertension; coexisting diabetes or other metabolic disorders; obesity; sleep disorders (obstructive sleep apnea); previously diagnosed or treated hyperuricemia/gout; dyslipidemia; thyroid disease; lack of precise information on the prior pharmacotherapy; anemia or thrombocytopenia; malignancies (solid tumors and hematological); chronic inflammatory diseases; active infections; chronic kidney disease with eGFR < 45 mL/min/1.73 m^2^; the presence of contraindications for ARBs and acetylsalicylic acid; the use of drugs which may affect the test results (including non-steroidal anti-inflammatory drugs, anticoagulants, phosphodiesterase type 5 inhibitors, nitrates); and female sex (due to interference with the menstrual cycle and hormonal contraception or hormone replacement therapy and childbearing age—a contraindication for use of ARBs). All patients underwent the cardiovascular risk stratification by measuring conventional biochemical markers in accordance with the guidelines of the European Society of Cardiology (ESC) [17].

**Figure 2:**
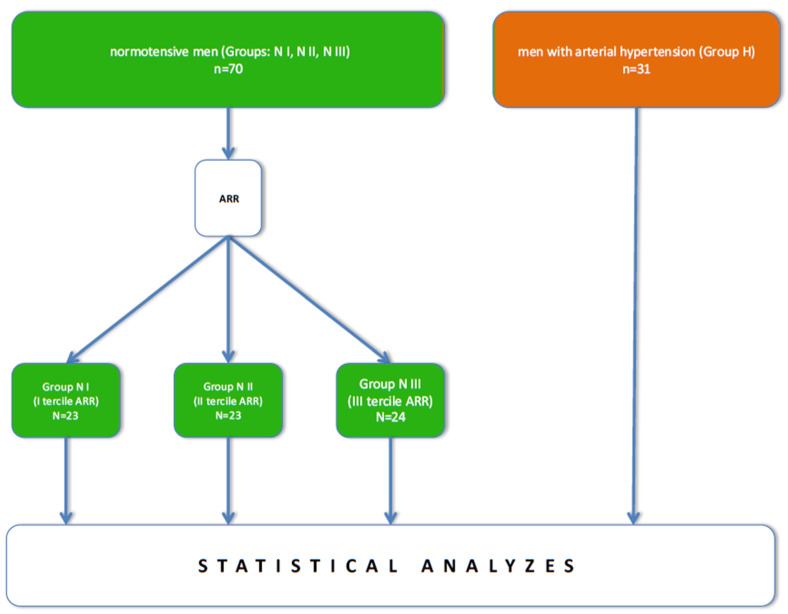
A flow chart—study design—schematic patients’ categorization. ARR—aldosteron to renin ratio.

The recruitment of male subjects for the study was preceded by a preliminary physical examination. Hypertension was diagnosed (the H group) or excluded (the N group). Subjects with normal blood pressure were divided into terciles, based on the value of the aldosterone-renin ratio (ARR) (N I, N II, N III, respectively) (Figure 2). The blood was collected from all patients twice: during the first recruitment visit (stage A), and on the fifth day, after four days of taking ASA (75 mg/24 h) (stage B). In the H group, the blood was drawn two more times: after two weeks of valsartan (80 mg/24 h) treatment (stage C), and one more time after four days of taking ASA (75 mg/24 h) together with valsartan (stage D).

The phenotype of endothelial function was assessed in terms of:

1. Vasodilatory function, which is dependent on the nitric oxide (NO) bioavailability resulting from the balance between its synthesis and degradation. The synthesis depends on the substrate availability (l-arginine), as well as on the concentration of the competitive inhibitors for the endothelial nitric oxide synthase (eNOS)—asymmetric and symmetric dimethylarginine (ADMA and SDMA, respectively). The degradation of NO depends mostly on oxidative stress leading to the peroxynitrite formation (ONOO^−^). Peroxynitrite—as an extremely unstable compound—leads to the nitration and S-nitrosylation of numerous proteins, and the nitrotyrosine (*N*-Tyr) is a marker of nitrosative stress. The bioavailability of nitric oxide was assessed also at the functional level by measuring the flow-mediated vasodilation (FMD) of the brachial artery.
2. Oxidative stress, measured as the lipid peroxidation, where malonyldialdehyde (MDA) is a marker, and nitrosative stress, as described above. Moreover, the antioxidative glutathione capacity was measured and expressed as the reduced-to-oxidized glutathione ratio (thiol index).
3. Inflammatory and angiogenic function regulated by oxidative stress, which was expressed by measuring the release of cell adhesion molecules (ICAM-1, VCAM-1), E-selectin, as well as of the vascular endothelial growth factor (VEGF).
4. Expression of the cyclooxygenase (COX) metabolic pathway, by measuring the arachidonic acid cascade end-products (thromboxane B2—TxB2, and prostacyclin—6-keto-PGF1 alpha). The parameters analyzed in this study are marked in red in Figure 1.

**Figure 3:**
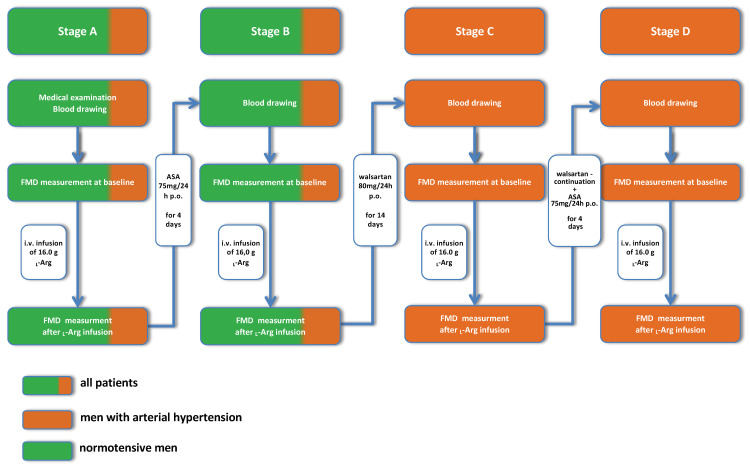
A flow chart—study design—schematic presentation of the study protocol. ASA—acetylosalicic acid; l-Arg—l-arginine; FMD—Flow-mediated dilatation.

After the blood was drawn, the vasodilatory reactivity of the brachial artery after reversible ischemia (FMD—flow mediated dilatation) was measured according to a published protocol [18]. Each group had the two FMD tests performed at each visit: the baseline, and after the administration of l-arginine (l-Arg) (Fresenius SE, Homburg, Germany). The cutoff point for ED was established as 10% of change in the brachial artery diameter in response to reactive hyperemia, when compared with the baseline value (Figure 3).

### 2.3. Blood Collection and Storage for Laboratory Assays

Medical examination of each subject was performed. Afterwards, at 7:30–09:00 a.m., fasting blood was drawn from the antecubital vein for subsequent laboratory testing in a commercially developed Sarstedt-Monovette (S-Monovette kit, Sarstedt AG & Co, Nuembrecht, Germany).

In order to avoid platelets activation, the blood was drawn without venous stasis. The following blood parameters were measured:

1. Plasma metabolites of NO pathway (ADMA, SDMA, l-Arg)—for subsequent high-performance liquid chromatography (HPLC) analysis;
2. Plasma prostanoids levels (TxB_2_ reflecting platelet COX-dependent activation, and 6-keto-PGF1 alpha reflecting endothelial antiaggregatory activation—for subsequent immunoenzymatica assays (EIA));
3. Plasma markers of oxidative stress (MDA—lipid peroxidation, GSH, GSSG, thiol index—GSH/GSSG—antioxidative capacity, and *N*-tyrosine—nitrosative stress from the eNOS uncoupling—marker of peroxynitrite formation);
4. Pro-inflammatory/angiogenic endothelial parameters (sICAM-1, sVCAM-1, PAI-1, sE-selectin, PAI-1, VEGF)—for subsequent immunoenzymatic assays (EIA);
5. Serum concentrations of total cholesterol (TCh), low-density lipoprotein (LDL), high-density lipoprotein (HDL), triglycerides (Tg), high-sensitive C-reactive protein (hsCRP), urea, creatinine, plasma glucose, thyroid stimulating hormone (TSH), sodium (Na^+^), potassium (K^+^), and uric acid (UA);
6. Aldosterone concentration and plasma renin activity.

The EDTA plasma was used to assess the levels of l-arginine (l-Arg), NG, NG-dimethylarginine (asymmetric dimethylarginine, ADMA) and NG, NG-dimethylarginine (symmetric dimethylarginine, SDMA), plasminogen activator inhibitor (PAI-1), and markers of oxidative stress. For determination of the prostanoid metabolites, plasma was collected in sodium citrate tubes, previously supplemented with 5 mM ASA solution (Sigma-Aldrich Sp. Z o.o., Poznań, Poland) to avoid the platelet activation. Blood was centrifuged and plasma-separated, and stored at −80 °C until analysis. The whole blood was also collected for EDTA to perform the complete blood count (CBC).

The concentrations of E-selectin, vascular endothelial growth factor (VEGF), intracellular adhesion molecule, soluble form (sICAM-1), vascular cell adhesion molecule 1, and soluble form (sVCAM-1) was measured in serum. The blood was also collected for a clot for analysis of the remaining biochemical tests in an accredited laboratory. After the blood was drawn, vasodilatory reactivity of the brachial artery after reversible ischemia (FMD) was measured in order to diagnose the endothelial dysfunction (ED).

### 2.4. Determination of Selected Parameters Characterizing the Endothelial Function

Plasma levels of l-Arg, ADMA, and SDMA were measured using high-performance liquid chromatography (HPLC), and precolumn derivatization was measured with o-phthaldialdehyde by a previously published method [19]. MDA levels were assessed using a lipoperoxidation marker in a colorimetric assay (LPO-586, BIOXYTECH, OXIS International, Inc., Beverly Hills, CA, USA) [20]. The thiol index (GSH/GSSG)—the ratio of reduced to oxidated glutathione—was measured using a colorimetric assay (GSH/GSSG-412 BIOXYTECH) described by Gunthenberg [21]. The 3-Nitrotyrosine ELISA Kit test (Cat. No. ABIN1113213) by Oxis, was used to determine 3′-nitrotyrosine, in accordance with the manufacturer’s instructions. Since both prostacyclin (PGI2) and thromboxane A2 (TXA2) are unstable compounds, their concentration was assessed indirectly by determining the level of non-enzymatic hydration products (prostacyclin 6-keto-PGF1α and TxB2). The commercial enzyme immunoassays 6-keto-PGF1α EIA Kit (Cat. No. 900-004) and Thromboxane B2 EIA Kit (Cat. No. ADI-900-002) from Enzo Life Sciences^®^ were used, respectively, following the manufacturer’s instructions.

Commercial enzyme immunoassays Quantikine ELISA Human sICAM-1/CD54 (Cat. No. DCD540), Quantikine Human sVCAM-1/CD106 (Cat. No. DVC00), Quantikine ELISA Human sE-Selectin / CD62E (Cat. No. DSLE00) by R&D Systems^®^, and Quantikine Human VEGF commercial enzyme immunoassay (Cat. No. DVE00) from R&D Systems^®^ were used to determine the concentration of sICAM-1, sVCAM-1, sE-Selectin, and VEGF, respectively, following the instructions provided by the manufacturer.

Aldosterone concentration was determined by quantitative radioimmunoassay with the RIA Aldosterone assay (Cat. No. IM1664) by IMMUNOTECH. Determination of plasma renin activity (PRA) was performed using the Renin Plasma Activity kit (Cat. No. R-EX-125) from ZENTECH, by radio-immunoenzymatic angiotensin I determination. The aldosterone-renin ratio (ARR) was calculated as the quotient of the aldosterone concentration and the renin activity in plasma.

The lipid profile, serum creatinine, estimated glomerular filtration rate (eGFR), fasting plasma glucose, urea, high-sensitivity C-reactive protein (hsCRP), thyrotropin (TSH), potassium, sodium, and uric acid were assessed in an accredited hospital laboratory using the following analyzers, respectively: Sysmex^®^ (XT-4000i); Siemens^®^ (The Dimension^®^ EXL™); Thermo Fisher Scientific^®^ (Konelab 20 Clinical Chemistry Analyzer); and Werfen^®^ (ACL TOP 550 CTS).

### 2.5. Measurements of the Endothelial Vasodilatory Function (FMD)

Ultrasound assessment of FMD in response to reactive hyperemia was performed according to the method described by Celermajer et al. [22] (ALOKA, SSD 5500 duplex model, (Willich, Germany) with a 7–14 MHz linear array transducer, and the ALOKA probe holder MP-AH 0001 for arm immobilization—in order to improve the accuracy of FMD measurement). Briefly, the brachial artery was visualized in B-mode, and the diameter was measured at baseline, and then during reactive hyperemia (induced by inflation and then deflation of a sphygmomanometer cuff around the limb, distal to the scanned part of the artery). The cuff deflation following an inflation period of 5 min to >30 mmHg above systolic blood pressure produces adequate hyperemia to allow flow-mediated dilatation. Peak artery diameter was measured, and FMD percentage was calculated using the following formula:

FMD% = 100 × d*max*/d*b*,

where d*max* equals maximum diameter of the artery, and d*b* equals the measured baseline diameter. Each measurement was obtained four times, and the mean values were calculated.

Additionally, the l-Arg solution at the dose of 16 g was given intravenously into the peripheral vein, and the test was repeated. Blood pressure (BP) measurements were performed at each step of the study protocol, and preceded the FMD measurement, before ischemia-inducing cuff inflation (A), and 10 min after the end of FMD measurement, following the reperfusion period and ultrasound analysis—measurement (B). The BP measurements were conducted each time on the second arm in order to prevent the effect of transient BP-measurement-induced ischemia-reperfusion on the obtained FMD measurements.

### 2.6. Measurements of Arterial Stiffness

The pulse wave velocity (PWV) assessment was performed using the Complior^®^ device (Artech Medical, France) and dedicated software.

The propagation velocity—PWV [m/s]—was calculated from the formula: PWV = D/T, (where D is the estimated distance between the two measuring points, and T indicates the time delay between two determined points—transit time).

The direct distances (dD) between sensors were measured by investigators, and wave transit time was estimated by the software as a delay between wave feet from different sensors. The velocity of 10 m/s was considered as the standard cut-off value for normal C-F (carotid-femoral) PWV, where 80% of the direct carotid-femoral tape-measured distance (dD) by the investigator was considered as the pulse wave traveled distance (D), and this value was entered into the Complior software for subsequent analysis.

All the measurements were performed in a quiet room, and patients lay in a supine position for at least 10 min before the examination in order to stabilize a heart rate and a blood pressure. After blood pressure measuring, four piezoelectric Complior transducers were placed on the skin over the carotid, radial, femoral, and dorsal pedis artery. After acceptable signal strength and quality had been obtained, pulse wave data was acquired, and computer calculation was performed. We have presented only C-F PWV measurement results, as others do not have proved prognostic value.

### 2.7. Statistical Analysis

Data is expressed as the mean ± SD. The differences between the means were assessed using Student’s *t*-test or the Mann–Whitney U test, depending on the distribution of the variables and the variety of variances. For comparison of more than two groups, MANOVA/ANOVA tests were used. If statistically significant differences were found, the post-hoc analysis was performed using Newman–Keuls tests. The results at the level of *p* < 0.05 were considered statistically significant. All calculations were performed using the Statistica 13.0 StatSoft^®^ program (StatSoft, Krakow, Poland).

## 3. Results

### 3.1. Baseline Demographic Characteristics

As shown in Table 1, pulse pressure and systolic pressure were significantly higher in the hypertensive (H) group when compared to the other ones. In male normotensive subgroups, the HDL increased, and LDL decreased with an increase in the aldosterone-to-renin ratio (ARR) (Table 1).

### 3.2. Selected Parameters of the RAA System

**Figure 4:**
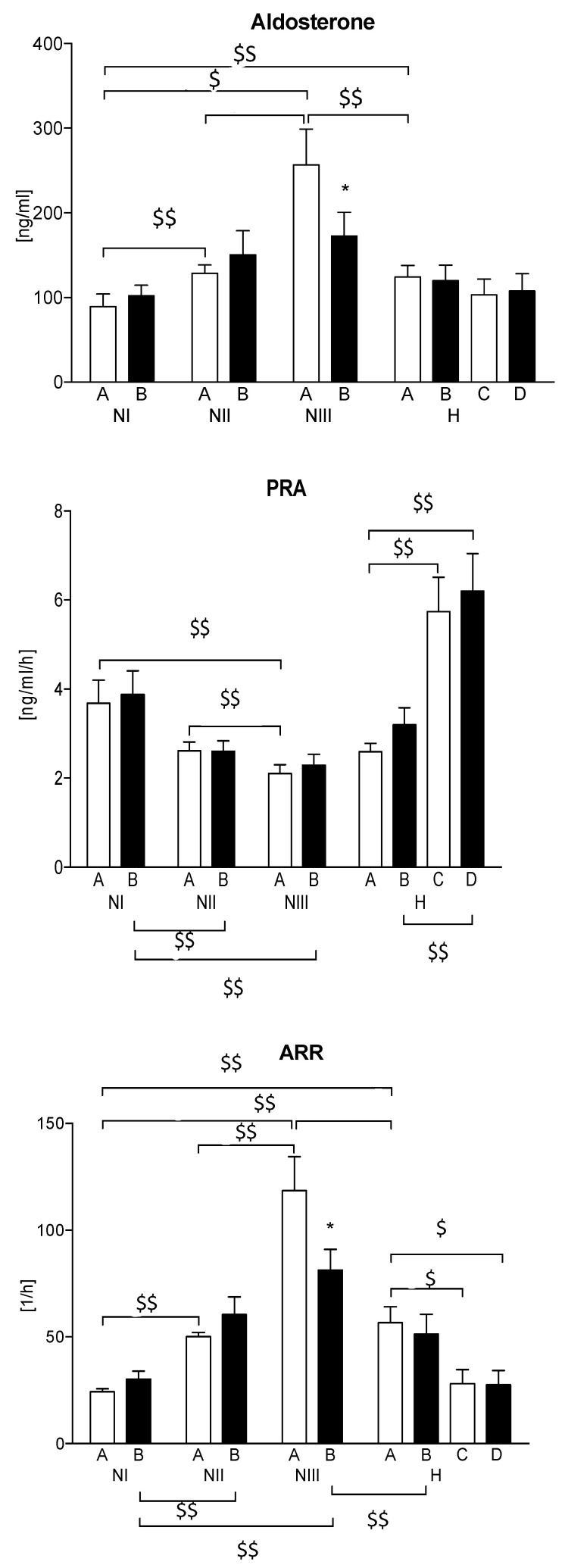
Assessment of the renin–angiotensin–aldosterone system. A—baseline; B—after ASA; C—after valsartan; D—after valsartan and ASA; PRA—plasma renin activity; ARR—aldosterone-to-renin ratio; * *p* < 0.05 A vs. B; $ and $$—*p* < 0.05 and *p* < 0.001 vs. another subgroup at the same step of study protocol, respectively.

In the normotensive men divided according to the level of the ARR index, the mean aldosterone level increased, and the PRA value decreased from NI to NIII, respectively. After ASA administration, a significant decrease in both ARR and aldosterone values in the NIII subgroup was observed. In hypertensive subjects, the baseline ARR and aldosterone were significantly lower when compared to the NIII subgroup. The use of valsartan resulted in a significant increase in PRA, leading to a decrease in ARR values. Administration of ASA did not result in changes in the PRA and ARR values in hypertensive subjects at baseline, nor after valsartan treatment. None of the pharmacological interventions significantly changed aldosterone levels in this group (Figure 4).

### 3.3. FMD

**Figure 5:**
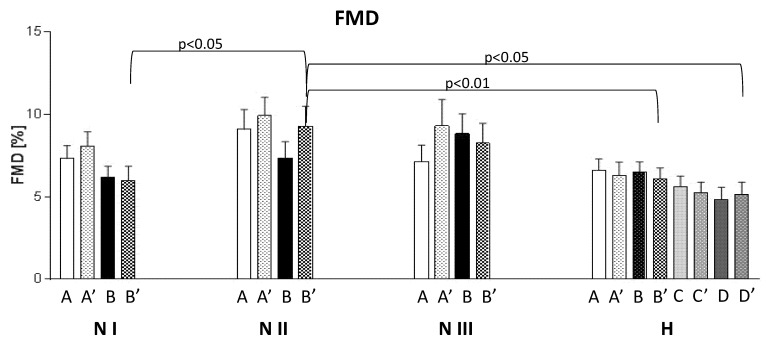
Flow-mediated dilatation (FMD). A—baseline; B—after ASA; C—after valsartan; D—after valsartan and ASA; FMD before (study A, B, C, D) and after l-Arg administration (study A’, B’, C’, D’).

There were no significant differences in FMD before and after the l-Arg administration, neither in the hypertensive nor in the normotensive subgroups, respectively. Significantly higher values of FMD were found after the use of ASA and l-Arg (B’) in the NII subgroup compared to both the NI and hypertensive (H) subjects. Noteworthy, in the H group, the use of valsartan and ASA did not affect the FMD values (Figure 5).

The blood pressure measurements were recorded, and the values obtained at the beginning, as well as at the end, of the study protocol are presented in Table 2. The protocol ended for normotensive men on stage B, whereas for hypertensives on stage D, following two weeks of valsartan treatment. The blood pressure values at the end of protocol vs. at the beginning did not change in normotensive subjects (NI, NII, and NIII), whereas both systolic and diastolic blood pressure have decreased in hypertensives. Nevertheless, the mean values in hypertensives were still within the “high normal” range.

### 3.4. Selected Parameters of the NO Bioavailability and Nitrosative Stress

The l-Arg level and the l-Arg/ADMA ratio were lower, and the ADMA level was higher in the H group compared to the normotensive subgroups. The significant differences in ADMA levels were observed between the H group and the NI and NIII subgroups, and between the NIII and NII subgroups. Interestingly, significantly higher baseline ADMA concentrations were observed in the NII compared to the NI subgroup. There was no effect of ASA on the l-Arg and l-Arg/ADMA ratio in any of the subgroups (Table 3). There were no significant differences in the values of these parameters, either after the administration of valsartan as monotherapy, or concomitantly with ASA (Table 4). SDMA concentration did not differ between groups (Table 3). However, a significant increase in SDMA was demonstrated following the valsartan administration (Table 4).

### 3.5. Markers of Oxidative Stress

**Figure 6:**
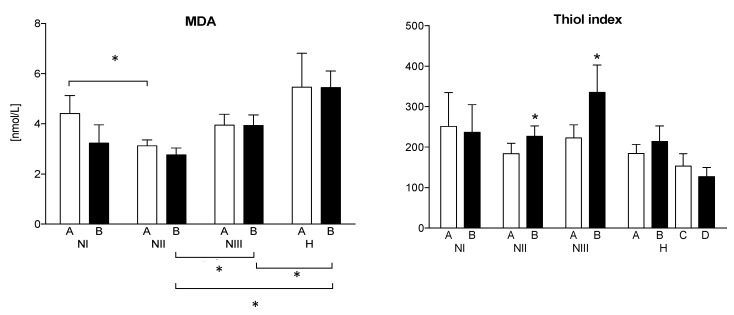
Markers of oxidative stress. A—baseline; B—after ASA; C—after valsartan; D—after valsartan and ASA; MDA—malondialdehyde. * *p* < 0.05 A vs. B as indicated.

Significant differences in the baseline MDA levels, reflecting lipid peroxidation, between the NI and NII subgroups were observed. Hypertensive subjects were characterized by the greatest MDA concentration. However, there were significant baseline differences between the H group and the NII and NIII subgroups. The use of ASA did not result in a significant decrease in MDA level. There were no significant changes in the values of the thiol index (antioxidant capacity) both at baseline and after administration of ASA. However, the use of ASA resulted in a significant increase in the thiol index in the NII and NIII subgroups (Figure 6). Management of hypertensive subjects with valsartan, as well as with both valsartan and ASA, did not change the thiol index. There were no significant differences between groups in the nitrative stress, as assessed by nitrotyrosine measurement (Table 3).

### 3.6. Markers of Inflammatory, Angiogenic, and Thrombogenic Function

#### 3.6.1. Endothelial Inflammatory Function—The Concentrations of sICAM-1 and sVCAM 1 and sE-Selectin

**Figure 7:**
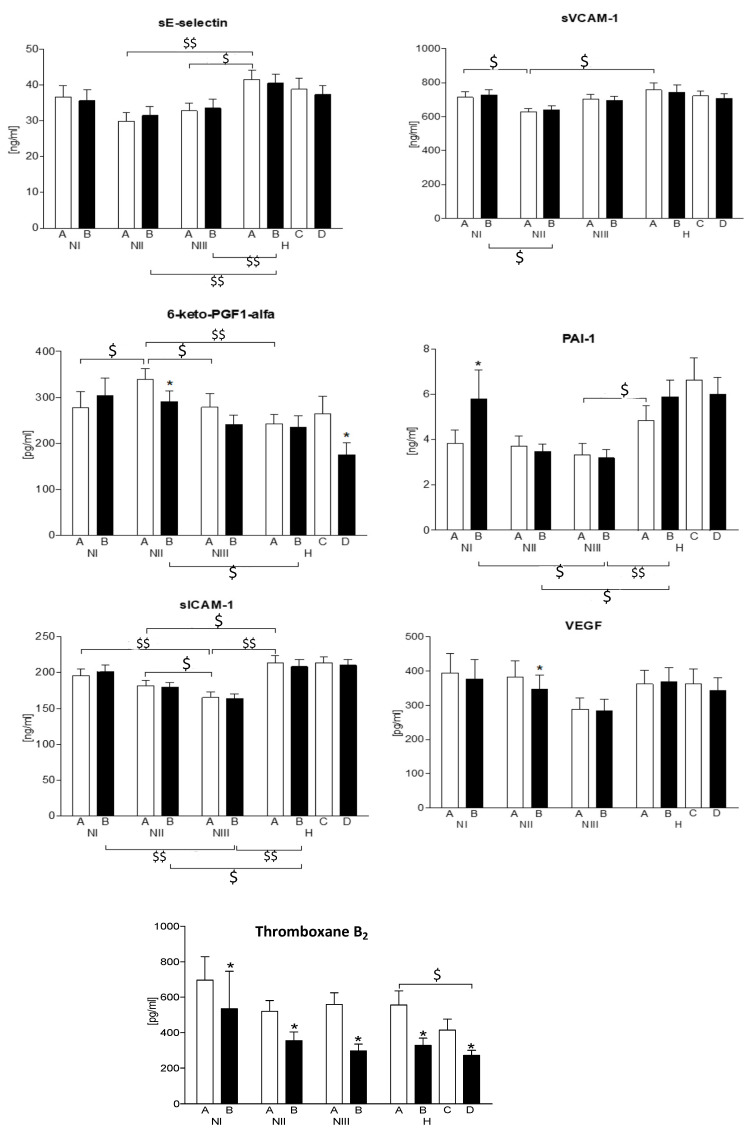
Parameters of inflammatory, angiogenic, and pro-thrombotic endothelial functions. A—baseline; B—after ASA; C—after valsartan; D—after valsartan and ASA. sICAM1—soluble intercellular adhesion molecule-1; sVCAM1—soluble vascular cell adhesion molecule; VEGF—vascular endothelial growth factor; PAI-1—plasminogen activator inhibitor-1. * *p* < 0.05 A vs. B and C vs. D; $ and $$—*p* < 0.05 and *p* < 0.001 vs. another subgroup at the same step of study protocol, respectively.

The concentration of sICAM-1 showed a downward trend, along with an increase in the ARR value, reaching the highest level in the NIII group. The baseline sICAM1 level was the highest in hypertensive subjects. None of the pharmacological interventions, including administration of ASA, exerted any effect on the sICAM1 levels in all analyzed subgroups. Similarly, in hypertensive subjects, the management with valsartan did not change the sICAM1 levels (Figure 7).

Similarly, the sVCAM1 level was the highest in hypertensive subjects, and lowest in the second ARR tertile of normotensive subjects. None of the pharmacological interventions (ASA), nor the use of valsartan in hypertensives changed the sVCAM1 levels (Figure 7).

The distribution of sE-selectin levels between the normotensive groups was similar to the one observed in sVCAM1, and hypertensive subjects were characterized by the highest sE-selectin concentrations. None of the pharmacological interventions (ASA, l-arginine), nor the use of valsartan in hypertensives changed the sVCAM1 levels, the same as in the case of sVCAM1, whereas the use of both, valsartan alone, and together with ASA decreased the sE-selectin levels and minimized the differences between the H group and the NII and NIII subgroups (Figure 7).

#### 3.6.2. Endothelial Angiogenic Function—Analyzing the VEGF Concentrations

Among normotensive subjects, the levels of sICAM-1 showed a downward trend, along with an increase in the ARR value, reaching the highest level in the NIII group. Administration of l-Arginine and ASA in NI, NIII, and H, as well as of valsartan in hypertensives did not exert any effect on the VEGF concentrations. The use of ASA caused a significant decrease in the VEGF level only in the NII group (Figure 7).

#### 3.6.3. Endothelial Thrombogenic Function—The PAI-1, 6-keto-PGF1 Alpha, and Thromboxane B6 Levels

Contrary to the baseline, thromboxane B2 concentration that did not differ between the groups, and the initial concentration of 6-keto-PGF1 alpha was the highest in the NII subgroup. The use of ASA, as a cyclooxygenase inhibitor, caused a significant decrease in the 6-keto-PGF1 alpha concentration in the NII subgroup. In group H, the use of valsartan and ASA also decreased the level of 6-keto-PGF1 alpha—nevertheless, a significant difference between its concentrations with the normotensive subgroup persisted (Figure 6). Administration of acetylsalicylic acid, as a cyclooxygenase inhibitor, resulted in a significant decrease in the platelet thromboxane synthesis in all the analyzed subgroups (A vs. B). Notably, in hypertensive subjects, the management with valsartan was associated with a decreasing trend of thromboxane, but without statistical significance (C vs. A, and D vs. B). Valsartan did not affect the inhibitory effect of ASA on the thromboxane synthesis in hypertensive subjects (D vs. C) (Figure 7).

The concentration of PAI-1 did not differ significantly between the normotensive subgroups. In hypertensive subjects, the initial PAI-1 values were significantly higher compared to the NIII subgroup. Nevertheless, the use of ASA caused a significant increase in PAI-1 concentration only in the NI subgroup. Valsartan had no effect on the PAI-1 levels in hypertensives.

#### 3.6.4. Effect of Pharmacological Interventions on Endothelial Inflammatory, Angiogenic, and Thrombogenic Function—Summary

Acetylsalicylic acid, a cyclooxygenase inhibitor, at a dose of 75 mg/24 h, limited the platelet synthesis of thromboxane B2, and its effect on the prostacyclin synthesis was minimal, and reached the statistical significance only in the NII from normotensives. Interestingly, in hypertensive subjects, ASA inhibited the 6-keto-PGF1 alpha synthesis, but only when valsartan was co-administered.

Management of hypertensive subjects with valsartan did not change the endothelial inflammatory and angiogenic activity, as assessed by the sICAM1, sVCAM1, sE-selectin, and VEGF levels, respectively. Notably, the management with valsartan was associated with a decreasing trend of thromboxane, but also a decrease in the prostacyclin synthesis.

## 4. Discussion

This is the first human-dynamic pharmacological study to precisely assess the effect of valsartan on the functional phenotype of the endothelium, and to verify its potential pleiotropic effects in young hypertensive subjects. As results from our study, treatment with valsartan exerted no significant effect on the vasodilatory endothelial properties, vascular microinflammation, nor the thrombotic or angiogenic activity. Nevertheless, valsartan was demonstrated to play an inhibitory role on both cyclooxygenase isoforms by limiting the thromboxane and prostacyclin synthesis.

The l-Arg was used in order to displace ADMA from the endothelial NO synthase active site (eNOS), and to increase the NO synthesis. To reduce NO degradation by reactive oxygen species, a cardioprotective dose of ASA was used, inhibiting the arachidonic acid cascade, and the synthesis of cyclic peroxides.

The increase in PRA in response to ASA may indicate a slight deterioration in renal perfusion. This relationship was mostly noticeable in the normotensive subgroup with the highest ARR values, and in the hypertensive subjects. As suggested by other authors, the inhibitory effect of ASA on the renal prostaglandin E2 (PGE2) synthesis may translate into a decrease in renal sodium loss, which could be associated with changes in the RAA system reactivity [23,24].

Similar baseline FMD values in all groups reflect comparable NO bioavailability. A negligible increase in the FMD values in the NII subgroup after l-Arg administration may suggest that the NO bioavailability was limited primarily by its reduced synthesis. Nishizak et al. [25] demonstrated that subclinical hyperaldosteronism causes endothelial dysfunction regardless of the blood pressure values. Our study was conducted on the young patients’ cohort, without clinically present atherosclerotic lesions, in which no changes in the FMD were observed either in response to l-Arg alone, nor in combination with ASA. This finding is somehow inconsistent with the results of another study [11], where an increase in FMD was observed after combined l-Arg and indomethacin treatment, indicating impaired NO bioavailability, both due to its decreased synthesis, and increased degradation. Contrary to our study, these differences occurred in cohort with primarily impaired FMD, and additionally, the pharmacological profile of indomethacin differs from the ASA. We did not confirm this effect in our study at a functional level of the endothelium (FMD). Although numerous reports point at a beneficial effect of valsartan on the endothelium [26,27,28], our results correspond with other studies, where valsartan’s effect was negative, as assessed by the forearm blood flow (FBF) measurement [29,30]. However, a beneficial effect has been noted with olmesartan, mostly due to oxidative stress reduction [31]. Notably, the FMD measurements do not reflect the microcirculation reactivity. Hence, additional tests, including peripheral arterial tonometry (PAT) or the laser doppler flowmetry (LDF), should be performed.

Greater MDA values in hypertensive subjects compared to normotensives with higher ARR values after COX blockade suggest the effect of reactive oxygen species from sources other than the arachidonic acid cascade. This is partially consistent with the studies by other authors showing an increase in lipid peroxidation in the course of essential hypertension [32,33]. Our results seem to contradict the conclusions of the study showing a reduction in lipid peroxidation because of COX blockade by indomethacin. However, in this study, the differences were found only in the subgroup with diagnosed endothelial dysfunction [11]. The increase in the thiol index after COX inhibition, observed in normotensive subjects with higher ARR, suggests a synergistic pro-oxidative effect of the RAA system and the COX pathway [34].

Higher levels of sE-selectin and sICAM-1 in hypertensives may indicate the endothelial inflammatory activation, which is consistent with the data showing increased expression of some polymorphic variants of E-selectin genes in the course of hypertension [35,36]. The lack of an ARR-dependent increase in the concentration of these parameters in normotensives suggests an effect of arterial hypertension, and increased vascular tone, rather than an aldosterone-dependent inflammatory reaction. The negative effect of valsartan on the concentration of these adhesive molecules, as well as sVCAM-1 contradicts the results of the Val_MARC clinical trial, where the use of valsartan (320 mg/day) led to a decrease in the sICAM-1 and sVCAM 1 levels [21].

In a few dose-defining studies, the antihypertensive effect of valsartan increased over a wide dose range (20–320 mg), and antihypertensive efficacy of the 80-mg dose was enhanced by doubling it up to 160 mg [37,38]. However, the effects of valsartan on selected parameters of endothelial function in the available literature were assessed for the mean dose of 80 mg OD. Hence, in order to be consistent with the data by other authors in terms of interpreted results, we have chosen this dosage in our experimental design. The decision was supported by the guidelines for the management of hypertension, where in young adults with mild hypertension, a single drug therapy at a middle dose was allowed at the beginning of treatment. Nevertheless, since numerous studies suggest that the optimal daily dose of valsartan ranges from 160 to 320 mg, which could be associated with better cardiovascular risk reduction [39], it can be assumed that greater doses of valsartan might be more effective in modifying endothelial function.

Higher PAI-1 levels and lower 6-keto-PGF1 alpha in hypertensive subjects suggests the endothelial pro-thrombotic activation. It could be the effect of either valsartan or ASA on PAI-1 levels. Interestingly, in subjects with diabetic nephropathy, Zhou G et al. [40] showed a beneficial effect of valsartan, which could be due to a different patients’ profile. A decrease in 6-keto-PGF1 alpha was also observed in our work after the concomitant use of ASA and valsartan. So far, the available literature shows an increase in the concentration of this parameter after valsartan treatment [41]. A decrease in platelet synthesis of thromboxane, and endothelial production of prostacyclin after valsartan management suggests an inhibitory effect on both COX isoforms, which requires further studies. In general, hypertensive subjects are characterized by decreased endothelial prostacyclin synthesis (as assessed by the 6-keto-PGF1 alpha concentration measurements), which might be partially attributable to the presence of endothelial inflammatory dysfunction in this subgroup (responsible for the earlier onset and progression of atherosclerotic burden in hypertensive subjects). The use of valsartan shows a hyper-additive synergistic effect with ASA in limiting the thromboxane production, which might be partially explained by changes in the oxidative stress, including lipid peroxidation, and as a result, decreased antioxidative capacity assessed by decreased thiol index. Decreased formation of cyclic peroxides resulting from lipid peroxidation may have favorable effects regarding the thromboxane. On the other hand, it also limits the prostacyclin synthesis via the COX metabolic pathway, which, in turn, limits the potentially pleiotropic effect of valsartan attributed to the TxB synthesis.

Comparable VEGF concentrations in all the groups suggests similar endothelial angiogenic activity regardless of the ARR index. There were also no significant changes after valsartan, which is consistent with the animal studies [42]. Interestingly, valsartan has been shown to exert local nephroprotective effects in the glomeruli through a tissue increase in VEGF expression responsible for the beneficial effects of monocytes and macrophages [43].

## 5. Conclusions

In conclusion, the administration of valsartan at a dose of 80 mg/24 h for two weeks had no significant effect on the NO bioavailability nor the endothelial vasodilatory, inflammatory, thrombotic, or angiogenic function. However, valsartan was shown to exert an effect on both cyclooxygenase isoforms by inhibiting the synthesis of thromboxane and prostacyclin. The clinical significance of this observation requires further research, and may partially explain the reduction of the beneficial effects of the angiotensin receptor blockers on the cardiovascular system with the simultaneous use of ASA.

## 6. Limitations

This study has the following limitations: (1) the young age of the patients, the male-only cohort, and the absence of cardiovascular comorbidities do not allow to extrapolate the results of this study on the whole burden of hypertensive subjects; (2) the minimal effective dose of valsartan was used in this study, which may not have been sufficient to produce the potential pleiotropic effects of the drug; (3) the assessment of the vasodilatory endothelial function based solely on the FMD method does not reflect the full spectrum of vascular reactivity, especially at the microcirculation level.

Therefore, in order to complete our knowledge of the pleiotropic effects of valsartan and other ARBs in subsequent studies, the protocol should be extended to a group of older patients with numerous comorbidities, and to the highest safe doses of the drug.

## Figures and Tables

**Table 1:** Baseline demographic characteristics and biochemical cardiovascular risk stratification.

	I Tercile ARR (NI)	II Tercile ARR (NII)	III Tercile ARR (NIII)	Arterial Hypertension (H)	*p* Value
	Mean ± SD	Mean ± SD	Mean ± SD	Mean ± SD	
N	23	23	24	31	
Age (years)	28.2 ± 1.21	26.83 ± 1.16	25.13 ± 0.87	27.37 ± 0.75	ns
HR (min^−1^)	70.5 ± 2.88	71.47 ± 2.26	70.78 ± 2.5	77.0 ± 3.0	ns
SBP (mmHg)	132.14 ± 4.59	127.78 ± 3.11	129.52 ± 2.51	145.85 ± 4.12	0.0009 *^#$^
DBP (mmHg)	80.71 ± 2.51	83.61 ± 2.09	84.04 ± 1.83	88.60 ± 2.65	ns
MAP (mmHg)	97.86 ± 3.14	98.33 ± 2.15	99.2 ± 1.85	107.68 ± 2.82	0.0106 *^#$^
PP (mmHg)	51.43 ± 2.48	44.17 ± 2.63	45.48 ± 2.04	57.25 ± 3.26	0.0044 ^#$&%^
Height (cm)	179 ± 1.94	180.68 ± 1.51	181.7 ± 1.17	180.61 ± 1.17	ns
Weight (kg)	82.88 ± 4.05	86.5 ± 1.99	85.39 ± 1.7	83.61 ± 2.88	ns
BMI (kg/m^2^)	25.76 ± 0.95	26.56 ± 0.64	25.88 ± 0.51	25.57 ± 0.77	ns
WHR	0.95 ± 0.02	0.94 ± 0.01	0.94 ± 0.01	0.95 ± 0.03	ns
PWV C-F (m/s)	9.64 ± 0.34	9.83 ± 0.34	9.79 ± 0.31	10.58 ± 0.37	ns
TCh (mg/dL)	186.18 ± 7.93	177.29 ± 11.38	174.16 ± 6.7	191.41 ± 7.47	ns
HDL-C (mg/dL)	47.09 ± 1.67	52.29 ± 2.13	56.26 ± 2.83	50.00 ± 2.03	0.0417 ^&^
LDL-C (mg/dL)	108.71 ± 7.6	97.89 ± 9.78	91.05 ± 6.99	116.90 ± 6.31	0.0485 ^#$^
TG (mg/dL)	154.62 ± 21.96	144.67 ± 15.98	134.53 ± 16.31	122.62 ± 13.32	ns
hs-CRP (mg/L)	2.57 ± 0.49	2.94 ± 0.36	3.2 ± 0.28	2.00 ± 0.60	0.0081 ^#$^
Glucose (mg/dL)	81.05 ± 1.68	86.09 ± 1.87	84.58 ± 2.75	91.61 ± 1.48	0.0012 *^#%^
Creatinine (mg/dL)	0.98 ± 0.02	0.98 ± 0.03	0.99 ± 0.03	1.05 ± 0.02	ns
eGFR (ml/min/1.73 m^2^)	128.83 ± 11.03	141.3 ± 5.55	137.5 ± 5.39	111.44 ± 7.54	0.0049 ^#$^
Uric acid (mg/dL)	6.08 ± 0.25	6.24 ± 0.28	5.94 ± 0.31	6.02 ± 0.22	ns
Urea (mmol/L)	31.57 ± 1.59	34.3 ± 1.85	32.5 ± 1.56	30.18 ± 1.23	ns
Sodium (mmol/L)	140.86 ± 0.57	141.91 ± 0.38	141.37 ± 0.41	139.93 ± 0.43	0.0121 ^#$^
Potassium (mmol/L)	4.01 ± 0.06	4.1 ± 0.07	4.0 ± 0.07	4.21 ± 0.06	ns
ARR baseline (A)	24.27 ± 1.14	50.17 ± 1.81	118.15 ± 15.1	56.51 ± 6.11	<0.0001 *^$&%^

HR—heart rate; SBP—systolic blood pressure; DBP—diastolic blood pressure; MAP—mean blood pressure; PP—pulse pressure; BMI—body mass index; WHR—waist–hips ratio; PWV C-F—Carotid-femoral pulse wave velocity; TCh—total cholesterol; HDL—high-density lipoproteins; LDL—low-density lipoproteins; TG—triglycerides; hsCRP—high-sensitivity C-reactive protein; eGFR—estimated glomerular filtration rate; ARR—aldosterone-to-renin ratio; results of post hoc paired analyses for unrelated groups: *—*p* < 0.05 for H vs. NI; ^#^—*p* < 0.05 for H vs. NII; ^$^—*p* < 0.05 for H vs. NIII; ^&^—*p* < 0.05 for NIII vs. NI; ^%^—*p* < 0.05 for NII vs. NI.

**Table 2:** Blood pressure measurements at the beginning and at the end of study protocol (for normotensives—A and B, for hypertensives A and D).

	I Tercile ARR (NI)	II Tercile ARR (NII)	III Tercile ARR (NIII)	Arterial Hypertension (H)	*p*
	Mean ± SD	Mean ± SD	Mean ± SD	Mean ± SD	
SBP A (mmHg)	132.14 ± 4.59	127.78 ± 3.11	129.52 ± 2.51	145.85 ± 4.12	0.0009 *^#$^
	vs.	vs.	vs.	vs.	
SBP end (mmHg)	130.00 ± 3.94	127.51 ± 4.91	128.26 ± 3.31	135.35 ± 3.80	ns
	*p* = ns	*p* = ns	*p* = ns	*p* = 0.00018	
DBP A (mmHg)	80.71 ± 2.51	83.61 ± 2.09	84.04 ± 1.83	88.60 ± 2.65	ns
	vs.	vs.	vs.	vs.	
DBP end (mmHg)	82.27 ± 2.11	82.50 ± 2.04	82.17 ± 1.65	82.5 ± 2.03	ns
	*p* = ns	*p* = ns	*p* = ns	*p* = 0.0115	

SBP—systolic blood pressure; DBP—diastolic blood pressure; A—measurement at the beginning of the study protocol; end—measurement at the last day of study protocol. Results of post hoc paired analyses for unrelated groups: *—*p* < 0.05 for H vs. NI; ^#^—*p* < 0.05 for H vs. NII; ^$^—*p* < 0.05 for H vs. NIII.

**Table 3:** Comparison of the nitric oxide bioavailability parameters between groups and within the same subgroup at particular steps of the study protocol.

	I Tercile ARR (NI)	II Tercile ARR (NII)	III Tercile ARR (NIII)	Arterial Hypertension (H)	*p*
	Mean ± SD	Mean ± SD	Mean ± SD	Mean ± SD	
l-Arg A (μmol/L)	63.06 ± 3.34	65.45 ± 3.34	60.70 ± 3.46	50.46 ± 3.70	0.0103 *^#$^
	vs.	vs.	vs.	vs.	
l-Arg B (μmol/L)	63.28 ± 3.94	69.12 ± 4.91	63.15 ± 3.31	51.83 ± 3.80	0.0101 *^#$^
	*p* = ns	*p* = ns	*p* = ns	*p* = ns	
ADMA A (μmol/L)	0.41 ± 0.02	0.46 ± 0.02	0.42 ± 0.02	0.51 ± 0.02	0.0009 *^#$%^
	vs.	vs.	vs.	vs.	
ADMA B (μmol/L)	0.43 ± 0.02	0.45 ± 0.02	0.40 ± 0.02	0.52 ± 0.03	0.0034 *^$%^
	*p* = ns	*p* = ns	*p* = ns	*p* = ns	
l-Arg/AMDA A	161.76 ± 12.77	147.49 ± 9.40	149.35 ± 9.06	102.81 ± 8.17	0.0004 *^#$^
	vs.	vs.	vs.	vs.	
l-Arg/AMDA B	152.48 ± 10.47	158.85 ± 13.84	166.71 ± 10.89	105.93 ± 8.46	0.0002 *^#$^
	*p* = ns	*p* = ns	*p* = ns	*p* = ns	
SDMA A (μmol/L)	0.46 ± 0.03	0.46 ± 0.02	0.50 ± 0.02	0.46 ± 0.02	ns
	vs.	vs.	vs.	vs.	
SDMA B (μmol/L)	0.49 ± 0.02	0.46 ± 0.02	0.53 ± 0.02	0.50 ± 0.03	ns
	*p* = ns	*p* = ns	*p* = ns	*p* = ns	
*N*-Tyr A (nmol/L)	48.90 ± 15.88	51.69 ± 20.17	50.68 ± 24.85	35.37 ± 5.94	ns
	vs.	vs.	vs.	vs.	
*N*-Tyr B (nmol/L)	60.66 ± 22.98	46.35 ± 14.80	86.58 ± 26.50	35.48 ± 7.31	ns
	*p* = ns	*p* = ns	*p* = ns	*p* = ns	

A—baseline; B—after ASA; l-Arg—l-arginine; ADMA—asymmetric dimethylarginine; SDMA—symmetric dimethylarginine; *N*-Tyr—nitrotyrosine. Results of post hoc paired analyses for unrelated groups: *—*p* < 0.05 for H vs. NI; ^#^—*p* < 0.05 for H vs. NII; ^$^—*p* < 0.05 for H vs. NIII; ^%^—*p* < 0.05 for NII vs. NI.

**Table 4:** Comparison of the nitric oxide bioavailability parameters in the hypertensive subjects (Group H) at particular steps of the study protocol.

	A	B	C	D	*p*
	Mean ± SD	Mean ± SD	Mean ± SD	Mean ± SD	
l-Arg (μmol/L)	50.46 ± 3.7	51.83 ± 3.8	45.97 ± 3.57	48.84 ± 3.44	ns
ADMA (μmol/L)	0.51 ± 0.02	0.52 ± 0.03	0.51 ± 0.03	0.50 ± 0.03	ns
l-Arg/AMDA	102.81 ± 8.17	105.93 ± 8.46	97.02 ± 9.79	103.17 ± 9.68	ns
SDMA (μmol/L)	0.46 ± 0.02	0.50 ± 0.03	0.52 ± 0.03	0.50 ± 0.03	*p* < 0.05
			*p* < 0.01 for C vs. A		
*N*-Tyr (nmol/L)	35.37 ± 5.94	35.48 ± 7.31	No data	No data	ns

A—baseline; B—after ASA; C—after valsartan; D—after valsartan and ASA; l-Arg—l-Arginine; ADMA—asymmetric dimethylarginine; SDMA—symmetric dimethylarginine; *N*-Tyr—nitrotyrosine. *p*-value for the overall effect in the variance analysis.

## Data Availability

The data presented in this study are available on request from the corresponding author.
